# The Contents of Trichloroacetic Acid-soluble Sulphydryl Compounds and Ascorbic Acid in the Liver of Rats Fed Aminoazo Dyes: the Effect of Continuous Feeding of Dyes in the Diet

**DOI:** 10.1038/bjc.1964.71

**Published:** 1964-09

**Authors:** J. Dijkstra, W. J. Pepler


### THE CONTENTS OF TRICHLOROACETIC ACID-SOLUBLE SULPHYDRYL COMPOUNDS AND ASCORBIC ACID IN THE LIVER OF RATS FED AMINOAZO DYES: THE EFFECT OF CONTINUOUS FEEDING OF DYES IN THE DIET

J. DIJKSTRA AND W. J. PEPLER

*From the National Chemical Research Laboratory, South African Council for Scientific and Industrial Research, Pretoria, and the Institute for Pathology, University of Pretoria, South Africa*

Received for publication March 16, 1964

IN the preceding paper (Dijkstra, 1964) it was reported that administration of a single large dose of aminoazo dyes to rats caused marked changes in the contents of trichloroacetic acid (TCA)-soluble sulphydryl (SH) groups and ascorbic acid in the liver. It was considered desirable to extend this investigation to continuous feeding of aminoazo dyes in the diet because the literature (see Dijkstra, 1964) suggested that the effect of continuous feeding might be different from that of a single dose of dye, and because it was hoped that parallel experiments with carcinogens and non-carcinogens might throw new light on the question of whether acid-soluble SH groups and ascorbic acid play a role in carcinogenesis. It was also of interest to compare the levels of SH compounds and of ascorbic acid in tumours induced by prolonged feeding of carcinogenic aminoazo dyes with the changes observed during the preneoplastic stage.

### MATERIALS AND METHODS

The diets were made by mixing 1 kg. of stock diet (Dijkstra and Joubert, 1961) with 24 ml. of olive oil or with 24 ml. of olive oil containing 2·66 m/moles of dye, i.e. 0·524 g. of aminoazobenzene (AB), 0·600 g. of 4-dimethylaminoazobenzene (DAB) and 0·637 g. of 2-methyl-DAB (2-MeDAB) or of 3′-MeDAB.

In a preliminary experiment, male albino rats (weight 187–214 g.) were fed for 1 to 8 days on a diet containing 3′-MeDAB. In the main experiment, male rats (weight 150–170 g. at the start of the experiment) were fed *ad lib.* on the above-mentioned diets. At intervals ranging from 2 days to 20 weeks, three rats on each of the five diets were killed and their livers analysed for TCA-soluble SH compounds and ascorbic acid. Parts of the liver of rats fed DAB or 3′-MeDAB for 10 and 20 weeks, were kept for histological examination.

The gross tumours used in these experiments were obtained from three male rats which had been fed on the 3′-MeDAB-containing diet for four months and then kept on the stock diet for six months. The amounts of tumour tissue obtained were 3·6 g. of cholangio carcinoma and 2·8 and 8·2 g. of hepatocellular carcinoma.

The other experimental procedures have been given in the preceding paper (Dijkstra, 1964).

### RESULTS

### *Dry matter content*

The dry matter content of the liver of rats fed the dyes, decreased to a minimum after two weeks' feeding and then increased (Fig. 1). Throughout the 20 weeks of the experiment, the dry matter content after feeding AB did not differ noticeably from the values of control rats receiving stock diet plus olive oil. After feeding 2-MeDAB, it was only slightly (average 0·4 per cent) lower, while after feeding the carcinogens DAB and 3′-MeDAB, it was on an average 1·8 and 2·1 per cent (relatively 6 and 7 per cent) below the control values.

This means that if the levels of SH compounds and ascorbic acid were calculated on a liver dry weight basis instead of on a wet weight basis, the levels would be relatively higher after about two weeks in the liver of all dye-fed rats and also relatively higher throughout the experiment in rats fed carcinogenic dyes. Because this did not change the interpretation of the results noticeably, the levels of SH compounds and ascorbic acid have been given on a liver wet weight basis.

The dry matter content of the tumours was not determined, but the amount of vacuum dried powder recovered from the residues of the TCA extraction after washing with alcohol, alcohol-ether, and ether, indicated that the dry matter content of the hepatocellular carcinomas was 68 and 70 per cent of that of the surrounding tissues, while that of the cholangio carcinoma was only 47 per cent of the dry matter content of the surrounding tissue.

### *SH levels*

Incorporation of olive oil in the diet had no effect on the level of TCA-soluble SH compounds in the liver, but feeding any of the dyes increased the level above normal (Fig. 2). An increase of about 25 per cent was noted even after 1 day's feeding with 3′-MeDAB (Table I). The increase in SH level during the first seven weeks of feeding a dye did not appear to be related to the carcinogenic activity,

TABLE I.—*The Contents of Trichloroacetic Acid-Soluble Sulphydryl and Ascorbic Acid in the Liver of Rats Fed 3′-MeDAB in the Diet*

| Number of days fed | Initial weight of rat | TCA-soluble SH compounds $\mu$moles/g. tissue (wet wt) | Ascorbic acid mg./g. tissue (wet wt) |
|---|---|---|---|
| 0* | 180 to 265 g. | 7·8 to 8·7 (mean 8·2) | 0·352 to 0·427 (mean 0·391) |
| 1 | 187 | 10·3 | 0·409 |
| | 212 | 10·3 | 0·371 |
| 2 | 188 | 11·0 | 0·444 |
| | 212 | 9·1 | 0·344 |
| 3 | 200 | 10·5 | 0·433 |
| | 206 | 10·5 | 0·445 |
| 4 | 199 | 9·9 | 0·379 |
| | 207 | 10·4 | 0·451 |
| 5 | 201 | 9·9 | 0·383 |
| | 218 | 10·9 | 0·405 |
| 8 | 195 | 10·0 | 0·436 |
| | 214 | 10·6 | 0·417 |

* 11 normal rats.

FIG. 1.—Dry matter content (percentage of wet weight) of the liver of rats fed on diets containing olive oil and aminoazo dyes.

The mean of three experimental values and the range, as well as the mean value for normal rats are indicated as follows :—

● = mean of three experimental values

● = range

——— = mean value for normal rats

– – – – = range

FIG. 2.—Content of TCA-soluble SH compounds (μmoles per g. of liver) in the liver of rats fed on diets containing olive oil and aminoazo dyes.

The mean of three experimental values and the range, as well as the mean value for normal rats are indicated as follows :—

● = mean of three experimental values

● (with range bar) = range

——— = mean value for normal rats

– – – – = range because it was greatest with the moderately carcinogenic DAB and least in the case of the strong carcinogen 3′-MeDAB.

On the other hand, the SH levels between 10 and 20 weeks were very much higher after feeding DAB or 3′-MeDAB than after feeding the non-carcinogens AB or 2-MeDAB. This coincided with histologically observed changes of the liver. The liver of rats fed DAB and 3′-MeDAB showed extensive proliferation of bile ducts and polyploidy of parenchymal cells with marked mitotic activity after 10 weeks. After 20 weeks they showed signs of hepatocellular carcinoma in parts of which bile duct differentiation was observed. There was also a cholangio carcinoma in the liver of one rat fed 3′-MeDAB for 20 weeks.

TABLE II.—*The Contents of Trichloroacetic Acid-Soluble Sulphydryl and Ascorbic Acid in Rat Liver Tumours Induced by Feeding 3′-MeDAB and in the Surrounding Liver Tissue*

| | TCA-soluble SH compounds, µmoles/g. tissue (wet wt) | | Ascorbic acid mg./g. tissue (wet wt) | |
|---|---|---|---|---|
| Tumour | Tumour | Surrounding tissue | Tumour | Surrounding tissue |
| Cholangio carcinoma | 4·4 | 8·5 | 0·138 | 0·236 |
| Hepatocellular carcinoma | 11·5 | 10·3 | 0·188 | 0·277 |
| Hepatocellular carcinoma | 11·7 | 9·4 | 0·233 | 0·281 |

Table II shows the TCA-soluble SH content of three liver tumours produced by feeding 3′-MeDAB and of the surrounding liver tissue. In the hepatocellular carcinomas the SH level was higher than in the surrounding tissue and in both it was higher than in normal rat liver. In the cholangio carcinoma the SH level was markedly below that in the surrounding tissue, where a normal level was found. When the dry matter content was taken into consideration, the high SH content of the hepatocellular carcinomas was even more striking, while the difference between the levels in the cholangio carcinoma and the surrounding tissue disappeared.

*Ascorbic acid level*

Incorporation of olive oil alone in the diet appeared to decrease slightly the ascorbic acid level in the liver (Fig. 3). This was still the case when the change in dry matter content was taken into consideration.

Feeding 3′-MeDAB did not result in distinct changes during the first 8 days (Table I), but on continued feeding the ascorbic acid level showed a marked increase above normal (Fig. 3). No distinct differences were observed between the slightly increased ascorbic acid levels in the liver of rats fed DAB and 2-MeDAB. The levels in rats fed AB were within the range of normal rats. The ascorbic acid content in the liver of rats fed the non-carcinogens AB and 2-MeDAB and the carcinogens DAB and 3′-MeDAB was on an average 0·04, 0·07, 0·07 and 0·13 mg. per g. of liver, respectively, above the values of the olive oil-fed control rats.

The ascorbic acid level of the 3 tumours was below that of the surrounding tissues, when calculated on a wet weight basis (Table II). On a dry matter basis

FIG. 3.—Content of ascorbic acid (mg. per g. of liver) in the liver of rats fed on diets containing olive oil and aminoazo dyes.

The mean of three experimental values and the range, as well as the mean value for normal rats are indicated as follows :—

● = mean of three experimental values

● = range

——— = mean value for normal rats

– – – – = range the differences disappeared. The level in the surrounding tissues was in all cases below normal. The observation of low ascorbic acid levels in tumours was in contrast to the results of Doi (1957) and of Briggs (1960).

### DISCUSSION

Continuous feeding of aminoazo dyes to rats caused increases of the content of TCA-soluble SH compounds in the liver. The increases observed during the first few days could not be simply correlated with those observed after a single dose, because continuous feeding of DAB caused the largest increase after only 2 days. Furthermore, continuous feeding of AB for a week also caused a rise of the SH levels in contrast to the behaviour after a single dose. Feeding 2-MeDAB and 3′-MeDAB never produced the large increases which were noticed after a single dose.

The extent of the increase in TCA-soluble SH content during the first 7 to 10 weeks of dye feeding could not be related to the induction of tumours, because there was no apparent difference between the effect of carcinogenic and non-carcinogenic dyes. The appearance of tumours was, however, accompanied by a distinct increase of the TCA-soluble SH content, which was not observed in rats fed non-carcinogenic dyes. It will be of interest to establish the nature of the SH compounds which are responsible for this late increase. Fiala and Fiala (1959) reported a late increase of sulphosalicylic acid-soluble SH compounds but a decrease of the glutathione content.

It has been suggested by Calcutt (1961) that a rise in total SH content is essential for carcinogenesis and that the total SH content decreases to low values before tumours appear. In contrast, the increase of the TCA-soluble SH content, reported here, was maintained until tumours appeared.

Furthermore, it has been shown that the TCA-soluble SH content was high in hepatocellular carcinoma and in the surrounding liver tissue, and that the low value in the cholangio carcinoma might be explained by a low dry matter content. These results may be contrasted to the report of Calcutt (1961) that rat hepatoma has both a lower glutathione and lower protein-SH content than normal rat liver.

Compared with the control values the ascorbic acid content of the liver was increased during feeding of aminoazo dyes, but no distinct pattern could be correlated with the induction or development of tumours. A decrease similar to that, which followed the initial increase after a single dose, was not observed until gross tumours occurred.

### SUMMARY

Feeding rats carcinogenic or non-carcinogenic aminoazo compounds in the diet caused a rise in the content of trichloroacetic acid-soluble sulphydryl compounds in the liver. During the initial 7 weeks, the increase was not characteristic for the carcinogenic process, but a further significant increase was found to accompany the development of tumours. The sulphydryl level was also high in aminoazo dye-induced hepatocellular carcinoma and its surrounding tissue, but was low in a cholangio carcinoma. The ascorbic acid content of the liver was more or less increased compared with that of control rats, but no changes were observed which were characteristic for the carcinogenic process. The ascorbic acid content was low in the tumours and their surrounding tissue.

The authors are indebted to Dr. H. M Schwartz for criticism and to Mr. J. J. Dreyer for kindly supplying the rats.

### REFERENCES

BRIGGS, M. H.—(1960) *Nature, Lond.*, **187**, 249.
CALCUTT, G.—(1961) *Brit. J. Cancer*, **15**, 673.
DIJKSTRA, J.—(1964) *Ibid.*, **18**, 608.
*Idem* AND JOUBERT, F. J.—(1961) *Ibid.*, **15**, 168.
DOI, G.—(1957) *Gann*, **48**, 243.
FIALA, S. AND FIALA, A. E.—(1959) *Brit. J. Cancer*, **13**, 136.


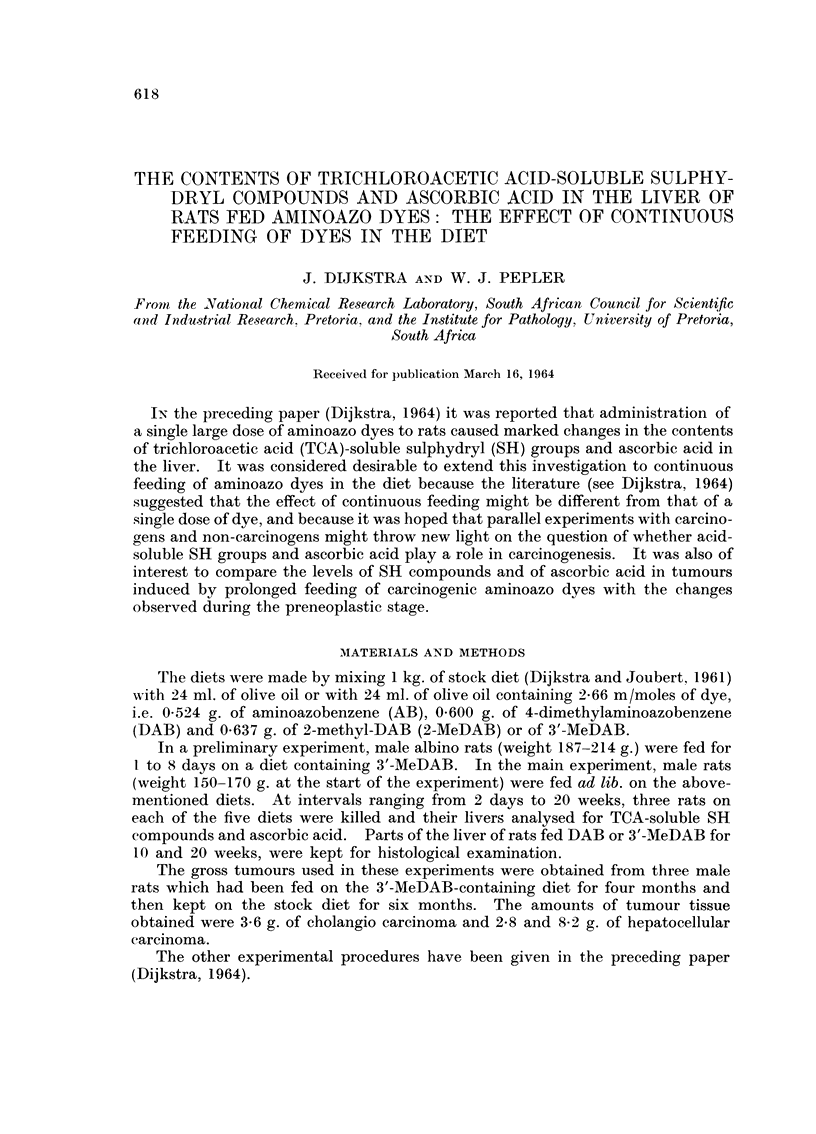

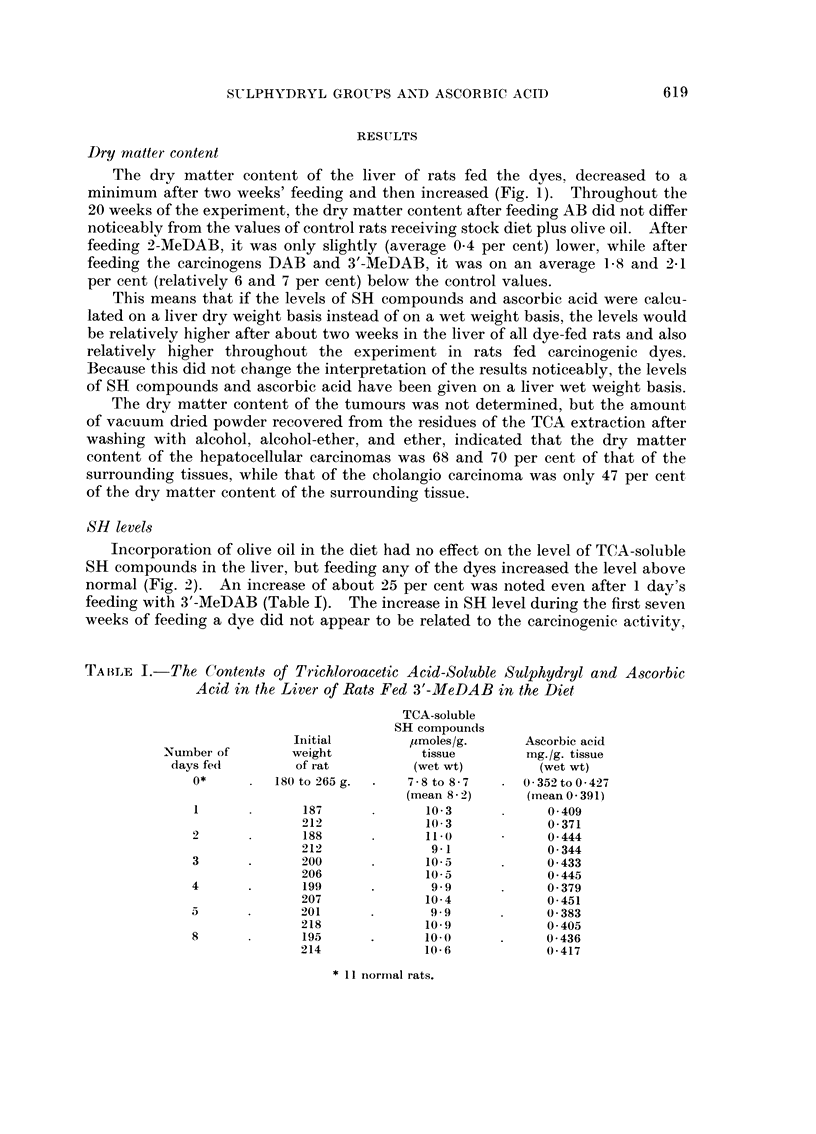

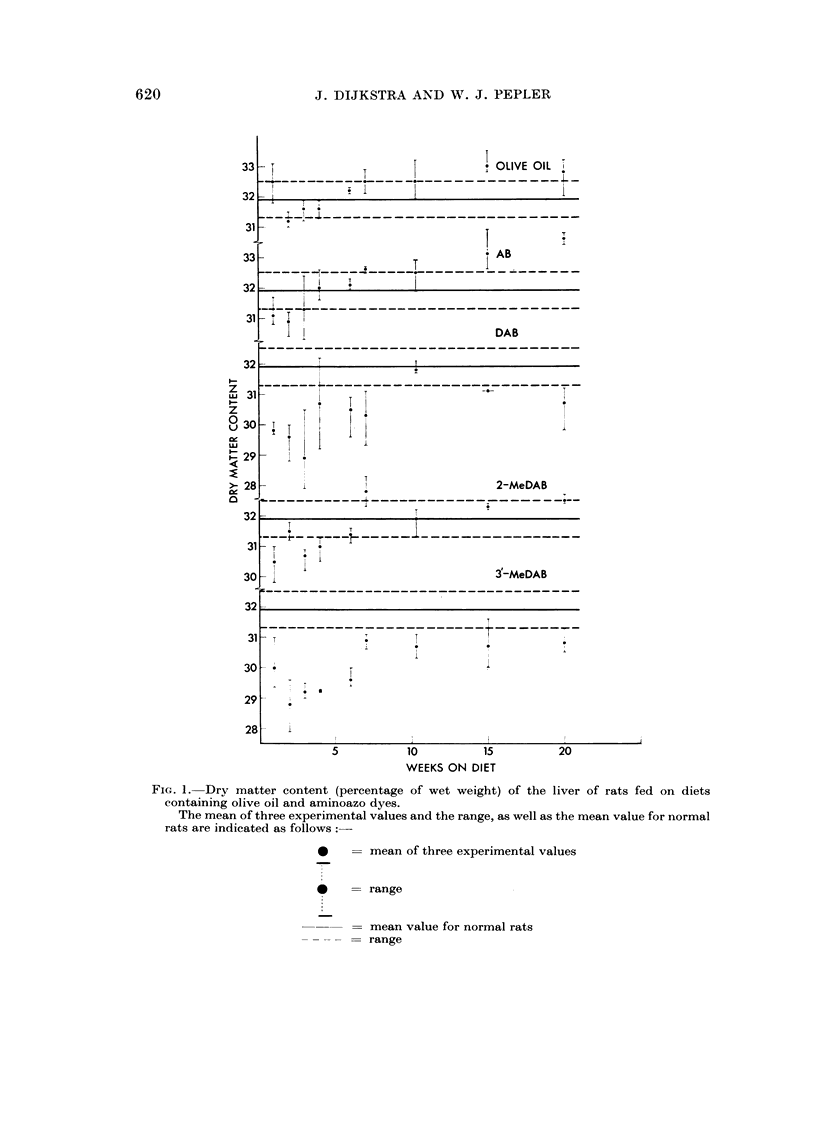

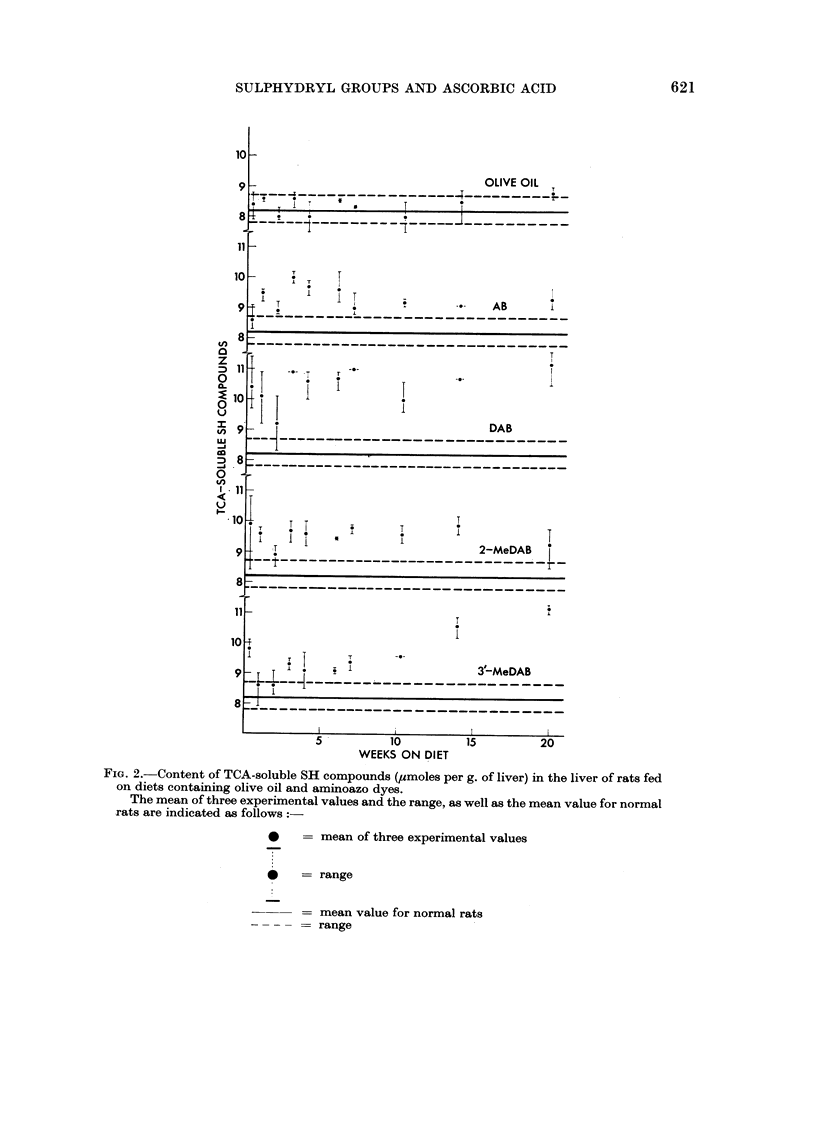

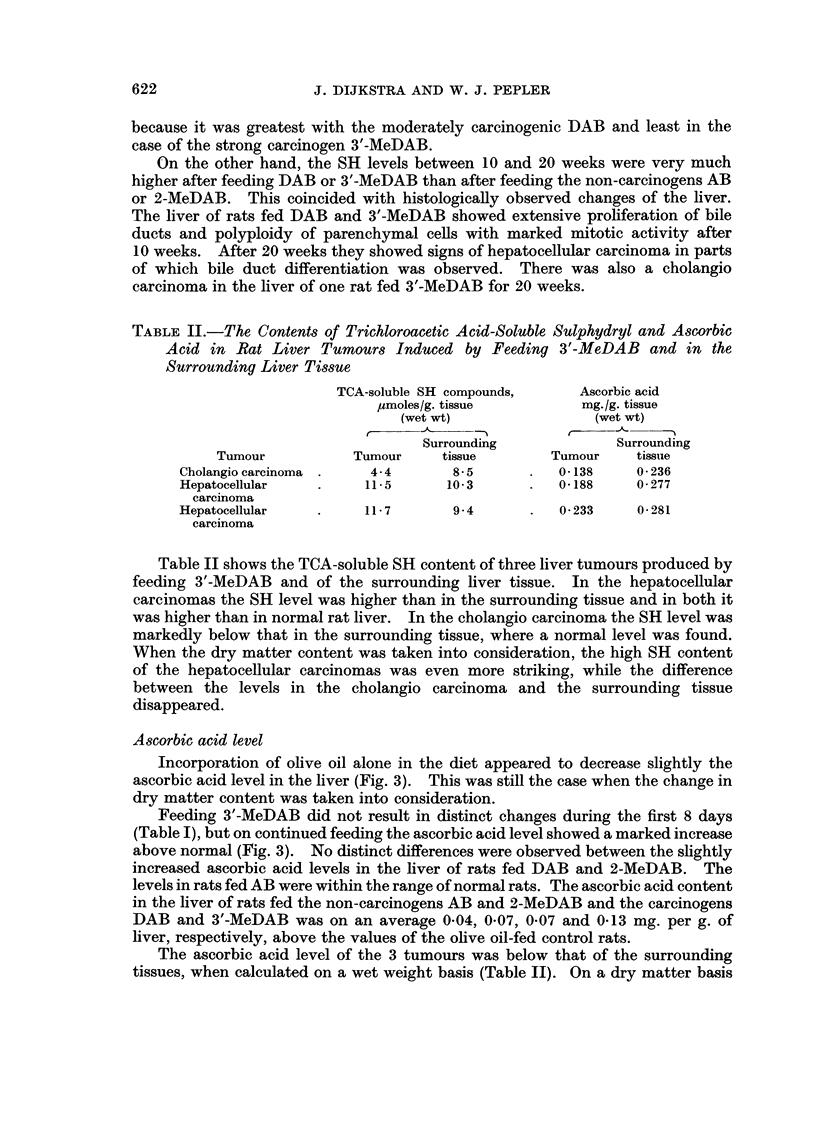

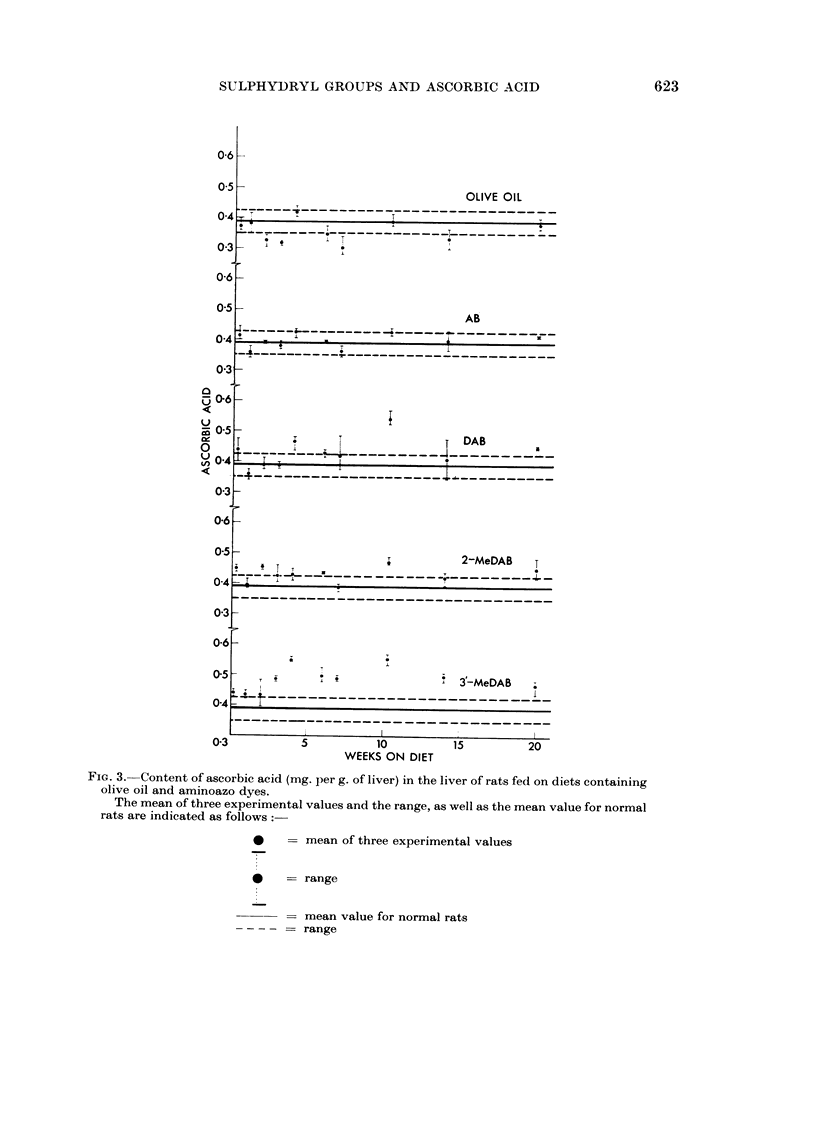

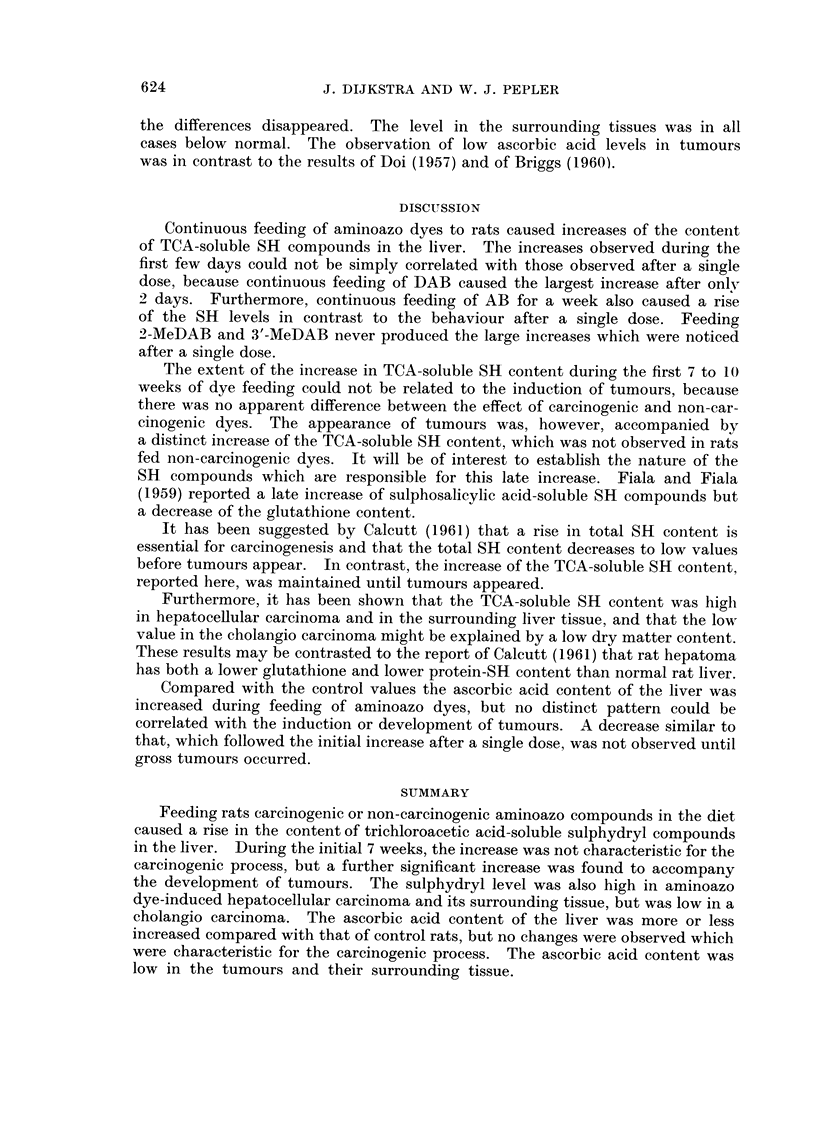

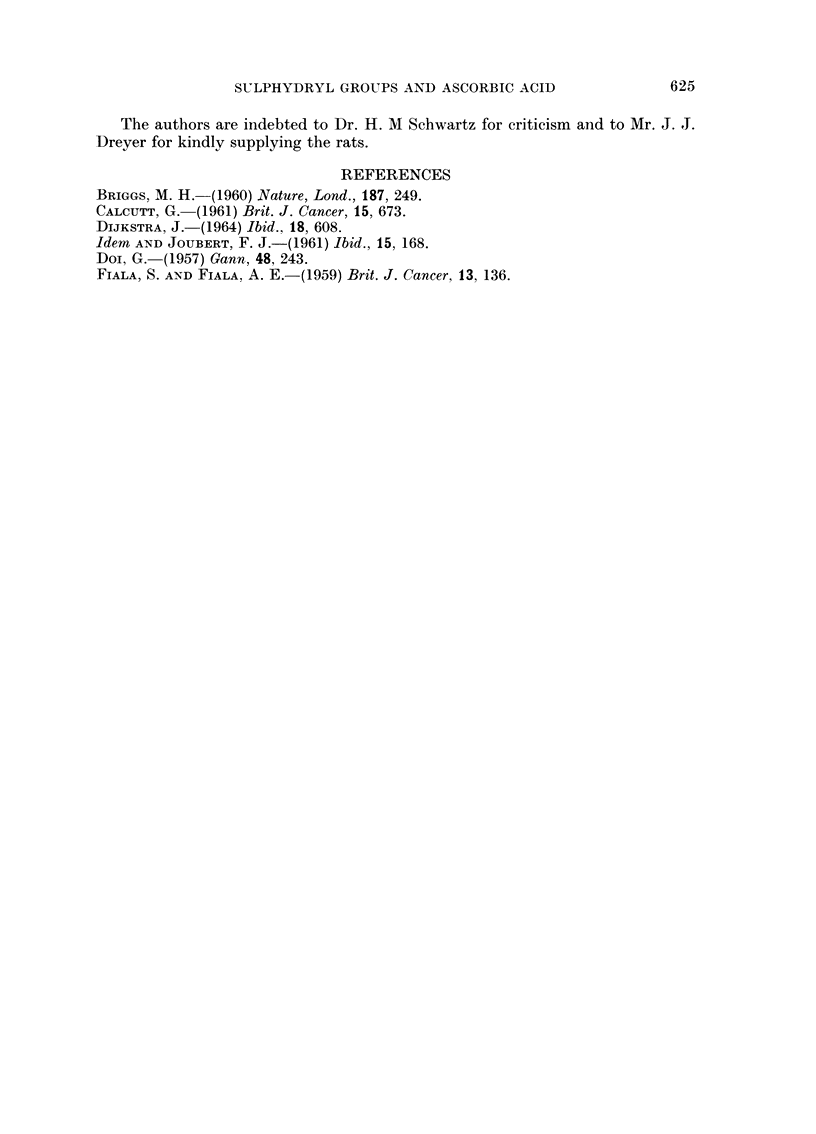


## References

[OCR_00463] BRIGGS M. H. (1960). Vitamin and coenzyme content of hepatomas induced by buter yellow.. Nature.

[OCR_00464] CALCUTT G. (1961). Sulphydryl groups and tumour induction by chemical agents.. Br J Cancer.

[OCR_00468] DOI G. (1957). Some chemical changes in the liver of rats fed p-dimethylaminoazobenzene. I.. Gan.

[OCR_00470] FIALA S., FIALA A. E. (1959). On the correlation between metabolic and structural changes during carcinogenesis in rat liver.. Br J Cancer.

